# Targeting lncRNA H19/miR-29b/COL1A1 Axis Impedes Myofibroblast Activities of Precancerous Oral Submucous Fibrosis

**DOI:** 10.3390/ijms22042216

**Published:** 2021-02-23

**Authors:** Cheng-Chia Yu, Yi-Wen Liao, Pei-Ling Hsieh, Yu-Chao Chang

**Affiliations:** 1 Institute of Oral Sciences, Chung Shan Medical University, Taichung 40201, Taiwan; ccyu@csmu.edu.tw (C.-C.Y.); rabbity0225@gmail.com (Y.-W.L.); 2 Department of Dentistry, Chung Shan Medical University Hospital, Taichung 40201, Taiwan; 3 School of Dentistry, Chung Shan Medical University, Taichung 40201, Taiwan; 4 Department of Anatomy, School of Medicine, China Medical University, Taichung 404333, Taiwan; plhsieh@mail.cmu.edu.tw

**Keywords:** oral submucous fibrosis, areca nut, *H19*, *miR*-*29b*, type I collagen

## Abstract

Oral submucous fibrosis (OSF) is known as a potentially malignant disorder, which may result from chemical irritation due to areca nuts (such as arecoline). Emerging evidence suggests that fibrogenesis and carcinogenesis are regulated by the interaction of long noncoding RNAs (lncRNAs) and microRNAs. Among these regulators, profibrotic lncRNA *H19* has been found to be overexpressed in several fibrosis diseases. Here, we examined the expression of *H19* in OSF specimens and its functional role in fibrotic buccal mucosal fibroblasts (fBMFs). Our results indicate that the aberrantly overexpressed *H19* contributed to higher myofibroblast activities, such as collagen gel contractility and migration ability. We also demonstrated that *H19* interacted with *miR*-*29b*, which suppressed the direct binding of *miR*-*29b* to the 3′-untranslated region of type I collagen (COL1A1). We showed that ectopic expression of *miR*-*29b* ameliorated various myofibroblast phenotypes and the expression of α-smooth muscle actin (α-SMA), COL1A1, and fibronectin (FN1) in fBMFs. In OSF tissues, we found that the expression of *miR*-*29b* was downregulated and there was a negative correlation between *miR*-*29b* and these fibrosis markers. Lastly, we demonstrate that arecoline stimulated the upregulation of *H19* through the transforming growth factor (TGF)-β pathway. Altogether, this study suggests that increased TGF-β secretion following areca nut chewing may induce the upregulation of *H19*, which serves as a natural sponge for *miR*-*29b* and impedes its antifibrotic effects.

## 1. Introduction

Over the past few decades, multiple oral precancerous lesions have been discovered, and oral submucous fibrosis (OSF) is one of them [1]. This chronic inflammatory disorder often results in restricted mouth opening and difficulty of cancer screening. Among various etiological factors, the consumption of areca nut has been indicated as a major causative factor in the development of OSF. Several studies have revealed that the constituents of areca nut (such as arecoline) elevate the secretion of transforming growth factor-β (TGF-β), which activates the transdifferentiation of myofibroblasts in the oral mucosa [2,3]. Under normal conditions, myofibroblasts are contractile cells expressing alpha-smooth muscle actin (*α*-*SMA*). Myofibroblasts help close wounds and produce the extracellular matrix (ECM), which includes fibronectin (FN1) and collagen, after injury [4]. In the final phase of tissue repair, the number of myofibroblasts decreases dramatically due to apoptosis. Nevertheless, failure of apoptosis and persistent activation of myofibroblasts often contribute to OSF [5].

Emerging evidence suggests that fibrogenesis, such as liver [6] or renal [7] fibrotic diseases, is regulated by multitiered epigenetic mechanisms orchestrated by noncoding RNAs, which lack protein-coding potential. There are diverse types of noncoding RNAs, including microRNAs (miRs; 21 to 24 nucleotides) and long noncoding RNAs (lncRNAs; longer than 200 nucleotides). It is known that miRNAs act through completely/partially complementary binding to the 3′-untranslated region (3′UTR) of their target genes. As for lncRNAs, various functions have been revealed, and one of them involves serving as competitive endogenous RNAs (ceRNAs) and interfering with miRNA binding, thereby depressing the effects of miRNAs [8]. In OSF, numerous studies have demonstrated that dysregulation of noncoding RNAs plays a significant role in the regulation of myofibroblast activities [9,10] and is associated with malignant progression [11]. Hence, a better understanding of the aberrant expression of noncoding RNAs may aid in the development of effective treatment for OSF.

The *H19* gene is an imprinted, maternally inherited transcript on chromosome 11p15.5 and encodes a 2.3 kb noncoding messenger RNA (mRNA) [12]. This lncRNA is tightly linked and coregulated with insulin-like growth factor 2. Furthermore, it is known that *H19* is abundant in numerous tissues from embryogenesis, but is downregulated postnatally. The aberrant expression of *H19* has been reported in various pathological conditions, such as malignant [13] or fibrotic [14] tissues. Multiple studies have shown that *H19* exerts its activities by acting as a molecular sponge. For instance, Lu et al. demonstrated that *H19* was significantly overexpressed in TGF-β-treated fibroblasts and bleomycin-induced lung fibrosis. Moreover, they revealed that *H19* interacts with miR-196a and is associated with the expression of alpha-1 chain of type I collagen (COL1A1) [15]. In addition to COL1A1, *H19* is also positively associated with α-SMA (ACTA2) expression in bleomycin-induced lung fibrosis [16]. Furthermore, Tang et al. showed that *H19* binds to antifibrotic *miR*-*29b* in alveolar epithelial cells, thereby inhibiting the expression of COL1A1 [16]. Given that the expression levels of *H19* [13] and *miR*-*29b* [17] were found to be significantly higher and lower in oral cancer tissues, respectively, we sought to investigate whether *H19* interacts with *miR*-*29b* and regulates the precancerous development of oral cancer.

In the current study, we evaluated the expression of *H19* in OSF tissues and examined its effects on myofibroblast activities. Subsequently, the relationship among *H19*, *miR*-*29b*, and COL1A1 was determined. We examined the functional role of *miR*-*29b* in the transdifferentiation of myofibroblasts and its association with several fibrosis factors. Furthermore, we investigated whether stimulation of buccal mucosal fibroblasts (BMFs) with arecoline activated the TGF-β pathway and affected the expression of *H19*. Altogether, these experiments were aimed at revealing the molecular mechanism underlying the pathogenesis of areca-nut-associated OSF.

## 2. Results

### 2.1. H19 Is Overexpressed in OSF Tissues and Associated with the Expression of Myofibroblast Markers

**Figure 1:**
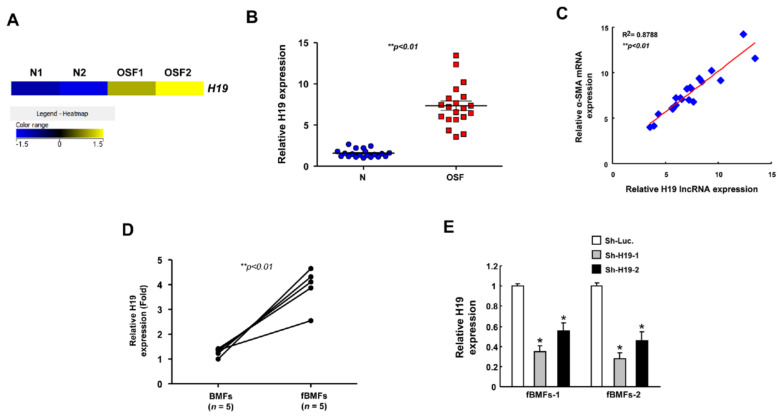
The expression of *H19* is aberrantly upregulated in oral submucous fibrosis (OSF) tissues. (**A**) Heatmap representation of differentially expressed genes between normal (*n* = 2) and OSF tissues (*n* = 2). ** *p* < 0.01 (**B**) qRT-PCR analysis showing lncRNA expression of *H19* in normal (*n* = 20; blue dots) and OSF tissues (*n* = 20; red dots). (**C**) Positive correlation between myofibroblast marker smooth muscle actin (SMA) and *H19* in OSF tissues (blue dots). (**D**) RT-PCR analysis of the expression level of *H19* in fBMFs (fibrotic buccal mucosal fibroblasts) and BMFs (buccal mucosal fibroblasts) extracted from OSF and normal counterparts, respectively; ** *p* < 0.01 compared with normal BMFs. (**E**) Knockdown efficiency of lentivirus-mediated short hairpin RNA (shRNA) targeting *H19* in two patient-derived fibrotic buccal mucosal fibroblasts (fBMFs). * *p* < 0.05 compared to sh-Luc (luciferase).

From a heatmap of RNA-sequencing results, we identified various differentially expressed genes, showing that *H19* was one of the upregulated lncRNAs in OSF with respect to normal specimens (Figure 1A). To validate this finding, qRT-PCR was conducted, showing that *H19* was overexpressed in OSF compared with normal tissues (Figure 1B). Moreover, we found that there was a positive correlation between *H19* and myofibroblast marker α-SMA (Figure 1C). These results suggest that the increased expression of *H19* may be associated with oral fibrogenesis. By using qRT-PCR, we also showed that the expression of *H19* was indeed elevated in patient-derived fibrotic buccal mucosal fibroblasts (fBMFs) compared to normal BMFs (Figure 1D). Hence, we sought to investigate its functional effect on myofibroblast activities and utilized the lentiviral-mediated approach to silence the expression of *H19* in fBMF populations isolated from two patients (Figure 1E).

### 2.2. Silencing H19 Inhibits Myofibroblast Activities

**Figure 2:**
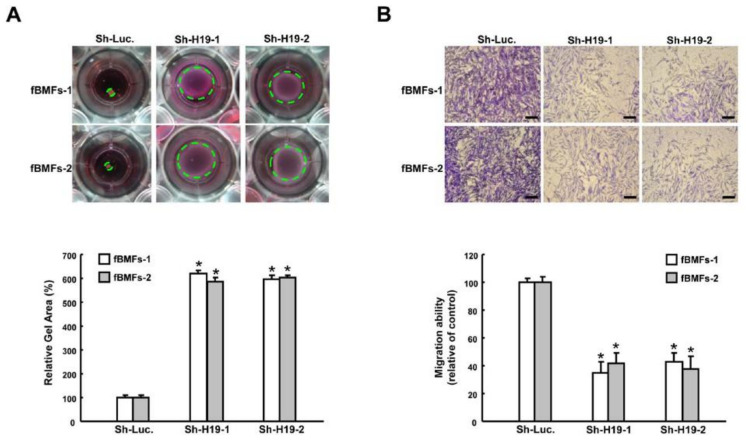
Downregulation of *H19* suppresses the phenotypes of myofibroblasts. (**A**) *H19*-silenced fBMFs were embedded into collagen gels. After 48 h, the contraction of gels was photographed, and the gel areas were calculated using Image J software v.1.8 (National Institutes of Health, Bethesda, Maryland, USA). (**B**) A Transwell migration assay was conducted to examine the effect of *H19* knockdown on migration ability after 24 h of incubation. Magnification, 100×. * *p* < 0.05 compared to sh-Luc.

The collagen gel contraction assay has been widely used to evaluate the differentiation of myofibroblasts, as a function of their role in wound closure and the fact that α-SMA expression augments their contractile capacity [4,18]. We observed that the size of the collagen gel matrix was increased in two fBMFs with short hairpin RNA (shRNA)-mediated silencing of *H19*, suggesting that the contraction activity was relieved when *H19* was lowered (Figure 2A). Another hallmark of myofibroblasts is their higher migratory capability. As shown in Figure 2B, knockdown of *H19* attenuated the migration ability in fBMFs, indicating that the upregulation of *H19* promoted the differentiation of myofibroblasts. As such, we aimed to elucidate the potential mechanisms underlying the profibrotic effect of *H19*.

### 2.3. H19 Promotes Myofibroblast Activation through the Suppression of miR-29b

**Figure 3:**
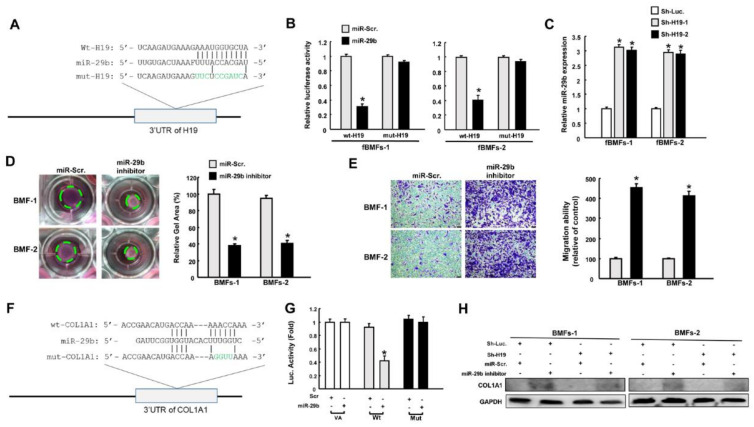
Repression of *miR*-*29b* by *H19* results in upregulation of type I collagen. (**A**) Schematic of *miR*-*29b* and the putative binding sequence, as well as the mutant sequence at the 3’-untranslated region (3′UTR) of *H19*. (**B**) Luciferase activity decreased when fBMFs were cotransfected with *Wt*-*H19* and *miR*-*29b* mimics. (**C**) Relative expression of *miR*-*29b* in *H19*-silenced fBMFs. Collagen gel contraction (**D**) and migration ability (**E**) of BMFs with *miR*-*29b* inhibitor or miR-Scr (scramble). Magnification, 100× (**F**) Schematic of *miR*-*29b* and the putative binding sequence in the 3′UTR sequence of Wt-COL1A1 and mutant sequence. (**G**) Luciferase activity was decreased when fBMFs were cotransfected with Wt-COL1A1 and *miR*-*29b* mimics. (**H**) Protein expression of type I collagen in BMFs with or without sh-Luc, *sh*-*H19*, miR-Scr, and *miR*-*29b* inhibitor. * *p* < 0.05 compared to miR-Scr.

The interaction between *H19* and *miR*-*29b* is implicated in bleomycin-induced idiopathic pulmonary fibrosis [16], and *miR*-*29b* has been revealed to function as an oncomir in oral cancer [16]. Therefore, we examined whether *H19* exerted its effect through targeting *miR*-*29b*. Figure 3A illustrates the complementarity between the 3′-UTR regions of *H19* and *miR*-*29b* to pinpoint the target sequence of *miR*-*29b*. Reporter plasmids containing either full-length (*Wt*-*H19*) or mutated (*mut*-*H19*) forms of the *miR*-*29b*-binding region were constructed and cotransfected with *miR*-*29b* mimics into fBMFs. We observed that the luciferase activity of *Wt*-*H19* vector was notably reduced when cotransfected with *miR*-*29b* mimics, whereas no significant change was present in the *mut*-*H19* vector in fBMFs (Figure 3B). In agreement with this finding, we found that the expression of *miR*-*29b* was upregulated in response to *H19* inhibition in fBMFs (Figure 3C). To verify if the decreased expression of *miR*-*29b* resulted in myofibroblast activation, an *miR*-*29b* inhibitor was added to normal BMFs. We demonstrated that both collagen gel contraction (Figure 3D) and migration ability (Figure 3E) were markedly enhanced.

Previously, *miR*-*29b* was shown to inhibit the transdifferentiation of C2C12 myoblasts into myofibroblasts via suppression of collagens [19]. Hence, we sought to evaluate the connection between *miR*-*29b* and type I collagen. We incorporated the point mutation in the 3′UTR to abolish the putative miR-29 recognition sequence of type I collagen transcripts (COL1A1) (Figure 3F). We showed that cotransfection with *miR*-*29b* mimics and Wt-COL1A1 suppressed reporter activity, but luciferase activity was not affected when cotransfected with the mutated form (Figure 3G). This finding indicates that *miR*-*29b* directly targeted COL1A1. Next, we demonstrated that the downregulation of *miR*-*29b* augmented the expression of type I collagen, whereas the suppression of *H19* reduced its expression. Moreover, the decreased expression of type I collagen via knockdown of *H19* was reversed when the *miR*-*29b* inhibitor was coadministrated in BMFs (Figure 3H and Figures S1A and S3, Supplementary Materials). These results suggest that *H19* regulated the protein expression of COL1A1 by counteracting the effect of *miR*-*29b*.

### 2.4. Overexpression of miR-29b Suppresses Myofibroblast Phenotypes and Expression of Fibrosis Markers

**Figure 4:**
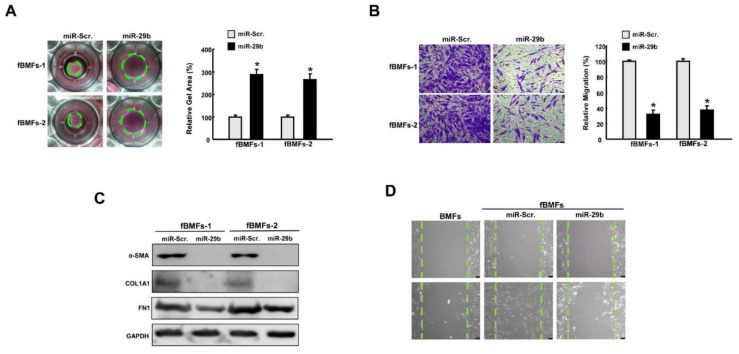
Overexpression of *miR*-*29b* mitigates the features of myofibroblasts. (**A**) Collagen gel contraction and (**B**) migration ability of fBMFs with miR-Scr or *miR*-*29b* mimics. Magnification, 100× (**C**) Relative expression of SMA, type I collagen, and fibronectin (FN1) in fBMFs with miR-Scr or *miR*-*29b* mimics. (**D**) Wound healing capacity of BMFs, fBMFs with miR-Scr, or fBMFs with *miR*-*29b* mimics. Magnification, 100×, * *p* < 0.05 compared to miR-Scr.

To further evaluate the functional role of *miR*-*29b* in myofibroblast activation, fBMFs with ectopic expression of *miR*-*29b* were subjected to various assays to examine the myofibroblast features and the expression of fibrosis markers. As expected, collagen gel contraction (Figure 4A) and migration capacity (Figure 4B) were diminished in *miR*-*29b*-overexpressing fBMFs. Furthermore, the protein expression levels of α-SMA, type I collagen, and fibronectin were all reduced (Figure 4C, Figures S1B and S4, Supplementary Materials). The scratch wound healing assay revealed that a higher closure rate was observed in fBMFs compared to normal BMFs, whereas overexpression of *miR*-*29b* in fBMFs delayed the repopulation of cells in the artificial gap (Figure 4D).

### 2.5. The Expression of miR-29b Is Downregulated in OSF Specimens and Negatively Correlated with Fibrosis Markers

**Figure 5:**
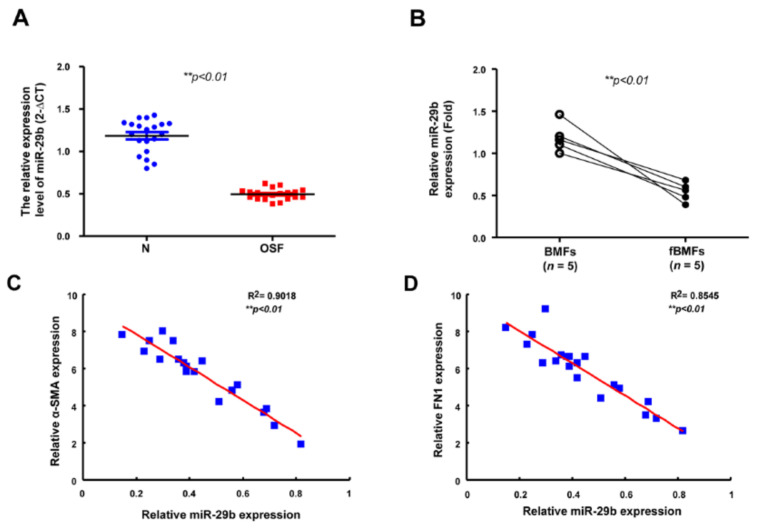
The expression of *miR*-*29b* is diminished in OSF and inversely associated with fibrosis markers. (**A**) Gene expression of *miR*-*29b* in OSF (red dots) and normal tissues (*n* = 20; blue dots). ** p < 0.01 (**B**) Relative expression of *miR*-*29b* in BMFs and fBMFs derived from OSF specimens (*n* = 5). ** p < 0.01 The expression of *miR*-*29b* was negatively correlated with (**C**) α-SMA and (**D**) FN1 expression in OSF tissues (blue dots) ** p < 0.01.

Subsequently, we verified the aberrantly reduced expression of *miR*-*29b* in OSF tissues (Figure 5A) and fBMFs derived from these specimens (Figure 5B). Moreover, we found that the expression of *miR*-*29b* was inversely related to the expression of myofibroblast marker α-SMA (Figure 5C). Similarly, the expression of *miR*-*29b* was inversely correlated to fibronectin, which was required for collagen matrix assembly (Figure 5D). Altogether, these results support the antifibrotic property of *miR*-*29b* in OSF and the association of the inadequate expression of *miR*-*29b* with oral fibrogenesis.

### 2.6. H19 Is Induced by Arecoline-Activated TGF-β Pathway

**Figure 6:**
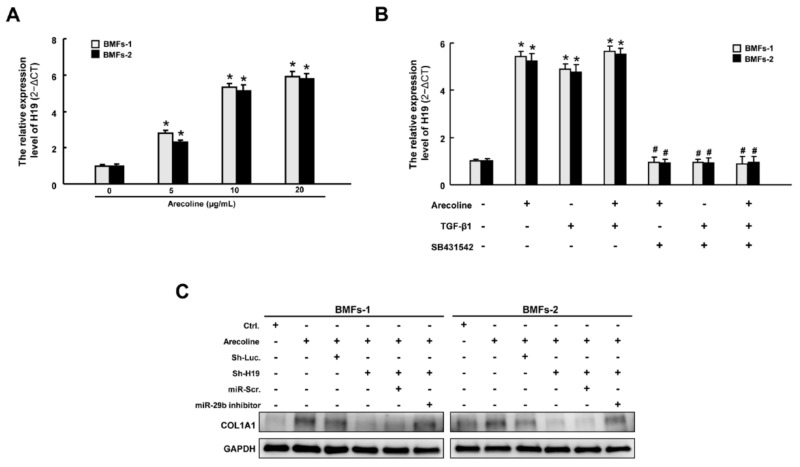
Arecoline increased the expression of *H19* through transforming growth factor (TGF)-β1. (**A**) The expression of *H19* was increased following arecoline stimulation in a dose-dependent manner. (**B**) Relative expression of *H19* in BMFs treated with arecoline, TGF-β1, or SB431542 (a specific inhibitor of the TGF-β type I receptor). * *p* < 0.05 compared to no treatment. # *p* < 0.05 compared to arecoline treatment. (**C**) Western blotting analysis describing the expression levels of COLA1 with indicated transfections.

**Figure 7:**
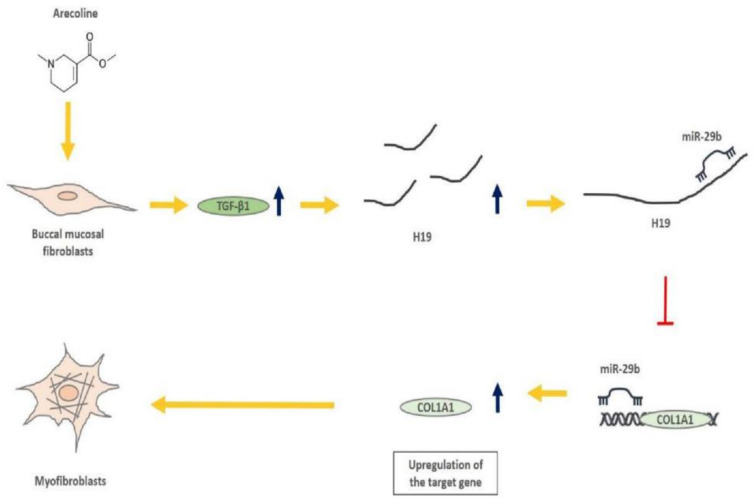
Possible mechanism for the areca nut-associated OSF. This study connects current knowledge and demonstrates that the upregulation of TGF-β by arecoline stimulation induces the expression of lncRNA *H19*, which sequesters *miR*-*29b* and impedes its binding of type 1 collagen 1. Moreover, suppression of the inhibitory effect of *miR*-*29b* on various fibrosis markers (such as type 1 collagen 1, α-smooth muscle actin, and fibronectin) results in transdifferentiation of buccal mucosal fibroblasts into myofibroblasts.

After elucidating that *H19* mediated OSF by interacting with *miR*-*29b* and suppressing its antifibrotic effect, we sought to ascertain the possible mechanism underlying the overexpression of *H19* in OSF tissues. It is well known that arecoline, a major alkaloid in areca nut, activates the TGF-β pathway, which is implicated in the transdifferentiation of myofibroblasts and the pathogenesis of OSF [1,2]. We demonstrated that the expression of *H19* was dose-dependently upregulated in BMFs following arecoline treatment (Figure 6A). Next, we showed that both arecoline and TGF-β1 induced the expression of *H19*. Nevertheless, administration of SB431542 (an inhibitor of the TGF-β type I receptor) abrogated the upregulation of *H19* caused by arecoline or TGF-β1, suggesting that the arecoline-induced *H19* was mediated by the TGF-β1 pathway (Figure 6B). To further connect the relationship among *H19*, arecoline, *miR*-*29b*, and OSF, we found that *miR*-*29b* suppression reverted the arecoline-stimulated *H19*-knockdown BMFs (Figure 6C and Figures S2 and S5, Supplementary Materials). Collectively, our results demonstrated that areca nut chewing elevated the expression of *H19* in BMFs through activation of TGF-β1. *H19* acted as a natural sponge for *miR*-*29b* and interfered with the direct binding of *miR*-*29b* to type I collagen, leading to oral fibrogenesis (Figure 7).

## 3. Discussion

Over the past few decades, various attempts have been made to explain the pathogenesis of precancerous OSF to delay or prevent its malignant progression. Several contributors are thought to be associated with impaired collagen homeostasis, including the TGF-β1 pathway. In addition to epithelial–mesenchymal transition (EMT) [20,21], our data demonstrated that *H19* is another downstream factor of TGF-β1, as the administration of SB431542 abrogated the elevation of *H19* induced by arecoline or TGF-β. *H19* has been investigated for years, and several studies have suggested that *H19* possesses both oncogenic and tumor-suppressive effects (see reviews [22,23]). In head and neck cancers, numerous studies have shown that *H19* was elevated, such as in tongue [24] or laryngeal [25] squamous cell carcinomas. As for its role in fibrogenesis, controversy also remains. Downregulation of *H19* was found in TGF-β1-stimulated hepatic stellate cells (HSCs) and liver tissues of CCl4-induced liver fibrosis. This study also showed that overexpression of *H19* inhibited the TGF-β1-induced proliferation of HSCs [26]. Nonetheless, *H19* seems to promote other fibrotic conditions, such as pulmonary fibrosis [15], cardiac fibrosis [27], or cholestatic liver fibrosis [28]. *H19* was found to impede the inhibitory action of EMT inducer ZEB1 by interacting with ZEB1 and preventing its binding to the promoter of epithelial cell adhesion molecule (EpCAM) [28]. Furthermore, *H19* acts to antagonize *YB*-*1*, a suppressor of COL1A1, through direct interaction [3]. It also serves as a sponge of *miR*-*196a* [4], *miR*-*140* [14], and *miR*-*29b* [16], thereby hampering their effects on the expression of *TGF*-*β1* or *COL1A1*. In agreement with these findings, we observed an upregulation of *H19* in OSF tissues, and the myofibroblast activities in fBMFs were reduced following *H19* repression. We confirmed the direct interaction between *H19* and *miR*-*29b* and showed that suppression of *miR*-*29b* enhanced the myofibroblast features in BMFs, whereas ectopic expression of *miR*-*29b* markedly lessened the fibrotic characteristics in fBMFs. Moreover, our data showed that *miR*-*29b* was able to directly bind to the promoter of COL1A1 in BMFs and was negatively associated with several fibrosis markers.

*MiR*-*29b* belongs to the *miR*-*29* family, consisting of *miR*-*29a*, *miR*-*29b*-*1*, *miR*-*29b*-*2*, and *miR*-*29c*. *MiR*-*29a* and *miR*-*29b*-*1* are located at chromosome 7q32, and *miR*-*29b*-*2* and *miR*-*29c* are located at chromosome 1q32. Increasing evidence suggests the antifibrotic role of *miR*-*29* in various tissues, as it inversely modulates several mRNAs encoding ECM proteins, such as COL1A1, COL1A2, COL3A1, elastin, fibrillin 1, and connective tissue growth factor [29,30,31]. In lung fibroblasts and mouse embryonic fibroblasts, it was demonstrated that the expression of miR-29 is negatively regulated by TGF-β/Smad3 [31,32]. Furthermore, it was demonstrated that TGF-β-induced loss of *miR*-*29b* expression occurs via Smad3 instead of Smad2 [32]. Qin et al. showed that Smad3 interacts with the *miR*-*29b* promoter and that *miR*-*29b* may be a transcriptional target of TGF-β/Smad 3 signaling [32]. In addition to being affected by Smad3, we further demonstrated that TGF-β-reduced *miR*-*29b* may be mediated by an increase in *H19*, which impedes the effects of *miR*-*29b*. In HSCs, the transfection of an *miR*-*29b* precursor was shown to blunt the increased expression of α-SMA, FN1, and platelet-derived growth factor receptor-β [29]. Similarly, overexpression of *miR*-*29b* inhibited TGF-β-stimulated collagen I and III (but not α-SMA) in mouse embryonic fibroblasts [32]. In line with these results, we demonstrated the antifibrotic role of *miR*-*29b* in fBMFs and showed that the downregulation of COL1A1 was due to the direct suppression of *miR*-*29b*. Nevertheless, there was a discrepancy in the effect of *miR*-*29b* on α-SMA. It was reported that no miR-29-binding sites were detected on the 3′UTR of α-SMA using multiple computational algorithms [19,32], and that the expression of α-SMA was not affected by *miR*-*29b* in mouse embryonic fibroblasts or renal proximal tubular cells [32]. However, α-SMA was found to be repressed following *miR*-*29b* overexpression in other types of cells, including C2C12 myoblast cells [19], mouse primary HSCs [29], and fBMFs. These findings suggest that *miR*-*29b* may not only inhibit fibrosis through directly targeting COL1A1, but also exert its antifibrotic property through other unidentified mechanisms. Further studies will be needed to elucidate the regulation of α-SMA by *miR*-*29b*. Whereas most studies have focused on the alteration of associated molecules following modulation of *miR*-*29b*, in the present study, we demonstrated that both *H19* and *miR*-*29b* had significant effects on myofibroblast phenotypes. The suppressed myofibroblast activities in both *H19*-silencing and *miR*-*29b*-overexpressing fBMFs may partially be due to the reduction in collagen I, as collagen and collagen-degradation peptides have been demonstrated to function as chemotactic stimuli for fibroblast migration [33,34].

## 4. Materials and Methods

### 4.1. Chemicals

Arecoline, SB431542 (a specific inhibitor of the TGF-β type I receptor), and collagen solution from bovine skin were purchased from Sigma-Aldrich (St. Louis, MO, USA). Arecoline stimulation was applied for the induction of myofibroblast transdifferentiation [2] and a collagen solution was used for collagen gel contraction analysis.

### 4.2. Tissue Acquisition

As for tissue collection, all procedures followed the tenets of the Declaration of Helsinki and were reviewed by the Institutional Review Board of Human Subjects Research Ethics Committee at Chung Shan Medical University, Taichung, Taiwan (approval number: CSMUH No. CS2-16142 (approval number: CSMUH No. CS2-16142; approval date: 10/05/2017). A total of 20 histological fibrotic mucosa or normal tissues were obtained from OSF patients or normal subjects recruited at the Department of Dentistry, Chung Shan Medical University Hospital.

### 4.3. Cell Culture

Upon approval by the Institutional Review Board of the Chung Shan Medical 364 University Hospital (approval number: CSMUH No: CS2-16142), two normal human BMFs isolated from normal controls, two fBMFs isolated from OSF tissues, and 293T cells were maintained in Dulbecco’s modified Eagle’s medium (DMEM) containing 10% *v*/*v* foetal bovine serum (FBS) (Thermo Fisher Scientific, Inc., Carlsbad, CA, USA) and 1% streptomycin/penicillin (Hyclone, Logan, UT, USA) as previously described [18].

### 4.4. RNA Sequencing

TRIZOL reagent (Invitrogen) was conducted to collect total RNAs for identification of the differential expression of the transcriptomes between normal and OSF tissues. The quality control of the RNA isolate from each sample was assured by the manufacturer of Genomics inc. before library construction. RNA-Seq libraries were sequenced paired-end on a HiSeq2500 (Illumina) according to the manufacturer’s instructions. Raw reads were processed using the Illumina CAS v.1.8.2 (Illumina, San Diego, CA, USA) to filter out the low-quality reads. The library preparations were sequenced on an Illumina platform and the paired-end reads. For the bioinformatics analysis, the sequence reads were aligned and calculated expression values according to FPKM (fragments per Kb of transcript per million mapped reads) for detection of discrepancies in the transcripts [5].

### 4.5. Quantitative Real-Time PCR

The TRIzol reagent was used for the isolation of total RNAs from cells, which were reverse-transcribed into complementary DNA (cDNA) using the SuperScript^®^ III First-Strand Synthesis System (Invitrogen Life Technologies, Carlsbad, CA, USA) following the manufacturer’s instructions for RT-PCR. The resulting first-strand cDNAs were used as templates for qPCR using an ABI StepOne™ Real-Time PCR System (Applied Biosystems, Foster City, CA, USA) and SYBR Green reagent with specific primers to anneal to the target sequences. GAPDH was used as the reference gene. [6]. The primer sequences used in this study were as follows: *H19*, 5′–CTCACCTTCCAGAGCCGATT–3′ and 5′–TAAGTTTCCGGGTCCGAACT–3′; *GAPDH*, 5′–CTCATGACCACAGTCCATGC–3′ and 5′–TTCAGCTCTGGGATGACCTT–3′.

### 4.6. Inhibition and Overexpression of H19

Lentivirus-mediated short hairpin RNA (shRNA) targeting *H19* was generated as follows: the pLV-RNAi vector was purchased from Biosettia Inc. (Biosettia, San Diego, CA, USA) for efficient delivery of RNA interference (RNAi) into cells via lentiviral transduction. The oligonucleotide sequence of lentivirus expressing shRNA against *H19* was synthesized and cloned into the pLV-RNAi vector, which was then cotransfected with a plasmid DNA mixture consisting of helper plasmids (VSVG and Gag-Pol) into 293T cells (American Type Culture Collection, Manassas, VA, USA) using Lipofectamine 2000 (LF2000, Invitrogen, Carlsbad, CA, USA). The target sequences for *H19* were listed as follows: *Sh*-*H19*-*1*: 5′- AAAAGCTTTCCTGTCTTTCCTTTATGGATCCATAAAGGAAAGACAGGAAAGC-3′; *Sh*-*H19*-*2*: 5′-AAAAGCTTTCCTGTCTTTCCTTTATGGATCCATAAAGGAAAGACAGGAAAGC-3.

### 4.7. Collagen Gel Contraction

A mixture of collagen gel and 2 × 10^5^ BMFs/fBMFs cells (with *sh*-*H19*, *miR*-*29b* inhibitor, or *miR*-*29b* mimic) was added to a six-well plate for polymerization. Then, the gels were detached from the well using a sterile pipette tip for measurement of gel contraction. The size of the gel was calculated using ImageJ software v.1.8 (National Institutes of Health, Bethesda, MD, USA).

### 4.8. Transwell Migration Assay

A two-chamber Transwell cell culture system with an 8 μm pore size polycarbonate membrane was used (Corning, Cambridge, MA, USA). 2 × 10^5^ BMFs/fBMFs were added to the upper chamber along with a serum-free medium. In the lower compartment, a serum-containing medium was used as the chemoattractant. After 48 h of incubation, cells on the other side of the insert were stained with crystal violet (Sigma-Aldrich, Sigma-Aldrich, St. Louis, MO, USA) prior to fixation. Cell numbers were counted from five different visual areas of 100-fold magnification under a microscope.

### 4.9. Luciferase Reporter Assay

The full length of *H19* was cloned and inserted into a pmirGLO plasmid (Promega, Madison, WI, USA) to generate the pmirGLO-*H19*-Wt reporter according to the manufacturer’s instructions, and the pmirGLO-*H19*-mut reporter was constructed via mutagenesis. For different experiments, fBMFs were cotransfected with pmirGLO-*H19*-Wt reporter, pmirGLO-*H19*-mut reporter, *miR*-*29b* mimic, or miR-Scr (scramble) using Lipofectamine 2000 reagent, followed by analysis of luciferase activity.

### 4.10. Western Blot Analysis

Protein samples were extracted from the BMFs or fBMFs. After denaturation, equal amounts of extracted proteins (30 μg) were separated by 10% SDS-PAGE with Tris-Glycine SDS running buffer and transferred with transfer buffer onto a nitrocellulose membrane (GE 446 Healthcare, Little Chalfont, Buckinghamshire, UK). Then, transferred membranes were incubated with BlockPRO blocking buffer (visual protein, energenesis biomedical co. ltd). After washing with TBST buffer (20 mM Tris, 150 mM NaCl and 0.1% Tween 20; pH 7.4) for 5 min 3 times, the transferred membranes were incubated with the primary anti-bodies against α-SMA (1:1000 dilution; Santa Cruz Biotechnology, Inc., Santa Cruz, CA, USA), COL1A1(1:1000 dilution; Santa Cruz Biotechnology, Santa Cruz, CA, USA), fibronectin (1:1000 dilution; Santa Cruz Biotechnology, Santa Cruz, CA, USA), or GADPH (1:5000 dilution; Thermo Fisher Scientific, Waltham, MA, USA). Then, transferred membranes were washed with TBST buffer for 5 min 3 times followed by incubation with the corresponding secondary antibodies. Developed chemiluminescence signals from catalyzed ECL substrate (Pierce Biotechnology) were detected using the LAS-1000 plus Luminescent Image Analyzer (GE Healthcare, Piscataway, NJ, USA). After the intensity of each band was measured by densitometry, the relative intensities were calculated by normalizing to GAPDH.

### 4.11. Wound Healing Assay

Confluent fBMFs were mechanically wounded by creating a straight line in the center of the well using a sterile 200 µL plastic pipette tip. The rate of the cells toward the denuded area was monitored and photographed at 0 and 48 h under a microscope.

### 4.12. Statistical Analysis

Three replicates of each experiment were performed. Data were expressed as the mean ± SD and analyzed using Student’s *t*-test. A *p*-value < 0.05 was considered statistically significant.

## 5. Conclusions

In conclusion, data from the present study delineated the potential mechanism underlying areca-nut-associated OSF. The activation of TGF-β signaling by chronic irritation due to areca nut led to the upregulation of *H19*, which impeded the *miR*-*29b*-mediated suppression of type I collagen, along with a lower inhibition of myofibroblast activities. Our results indicate that targeting the *H19*/*miR*-*29b* axis may be an effective approach to relieve the symptoms of OSF.

## Data Availability

Not applicable.

## Supplementary Materials

The following are available online at https://www.mdpi.com/1422-0067/22/4/2216/s1.
