# Supplementary material for: Targeting lncRNA H19/miR-29b/COL1A1 Axis Impedes Myofibroblast Activities of Precancerous Oral Submucous Fibrosis
Source: Int J Mol Sci. 2021 Feb 23;22(4):2216. doi: 10.3390/ijms22042216 (PMC7926971; doi:10.3390/ijms22042216)
Supplement: Supplementary file 1 [file ijms-22-02216-s001.pdf]

Supplementary Figure The relative level of indicated protein expression was normalized against GAPDH and the control was set as 100%. Optical density values represent the mean ± SD. Data shown here are the mean±SD of three independent experiments.

Suppl. Figure 1

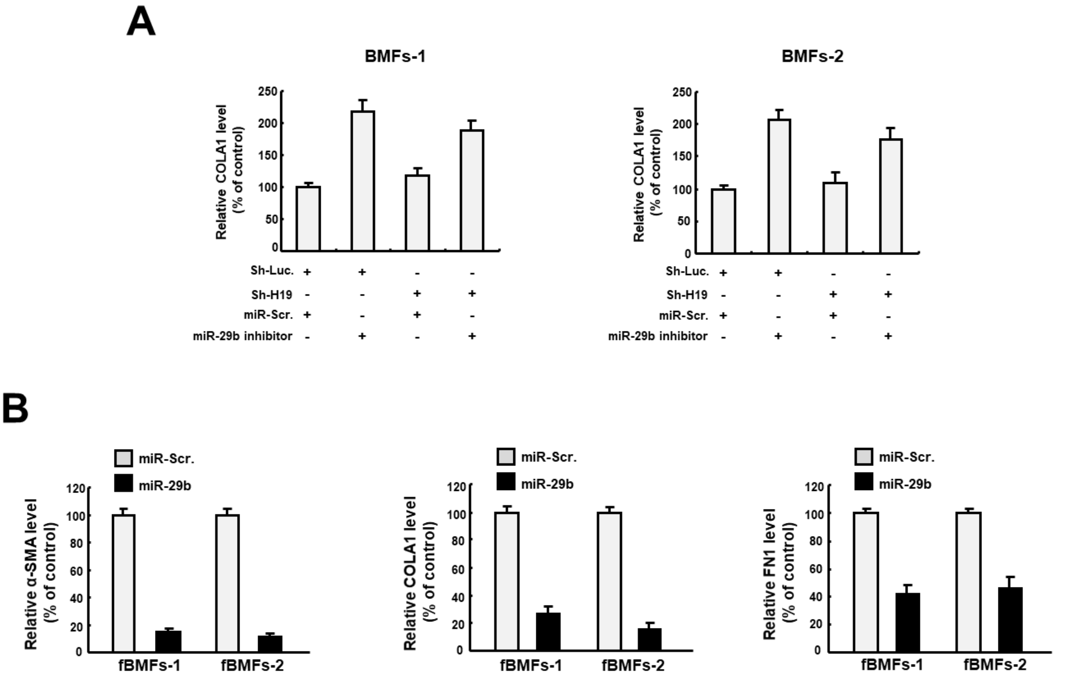

Suppl. Figure 2

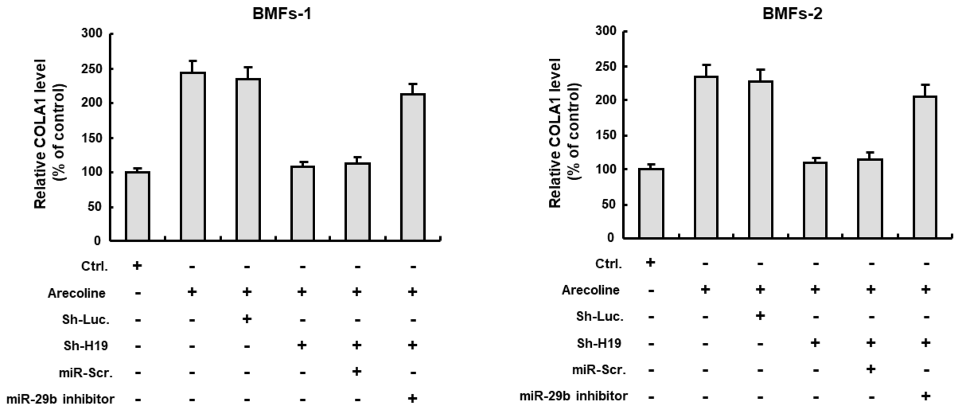

Suppl. Fig. 3

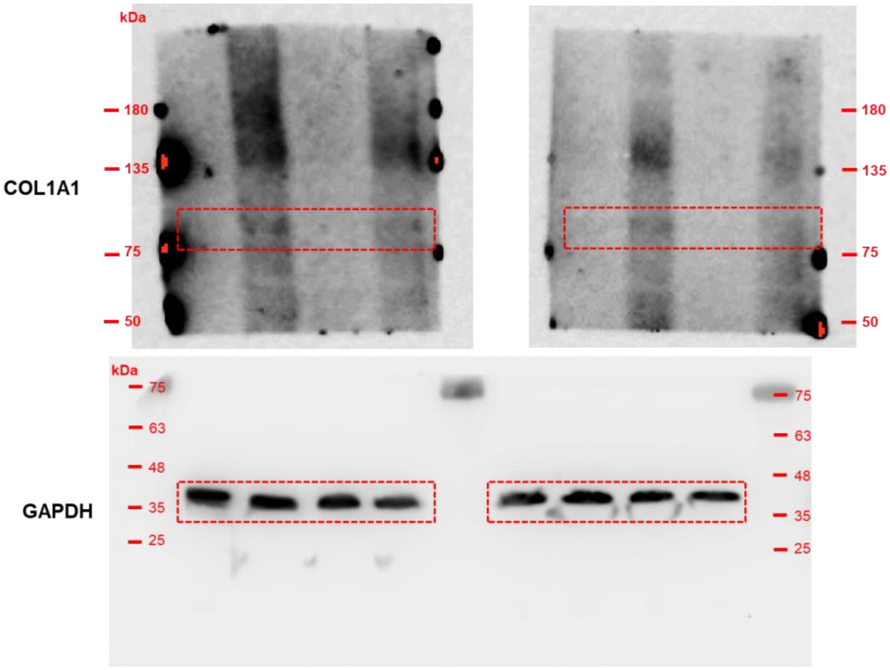

Supplementary Figure 3. Original immunoblotting data for Fig. 3H

Suppl. Fig. 4

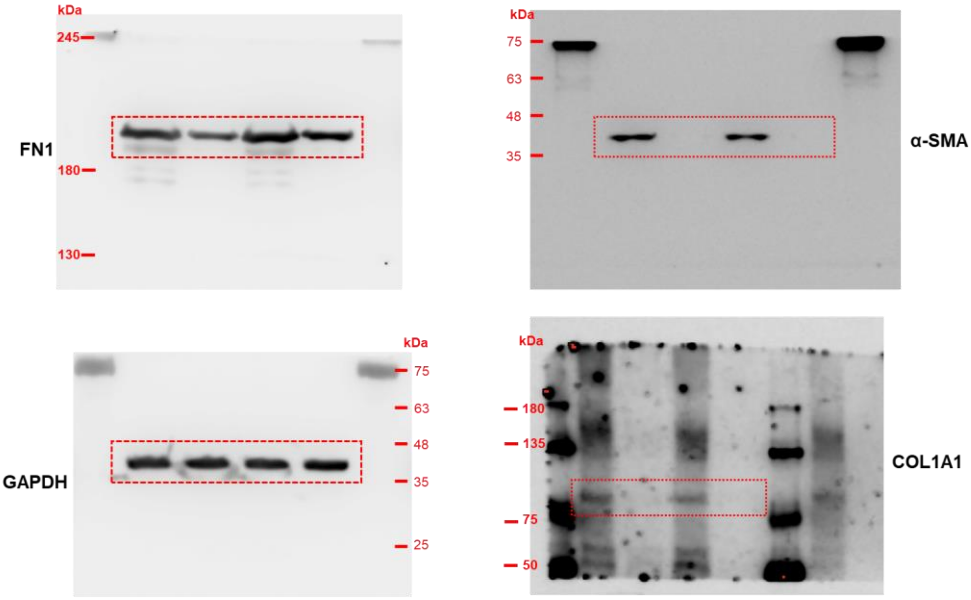

Supplementary Figure 4. Original immunoblotting data for Fig. 4C

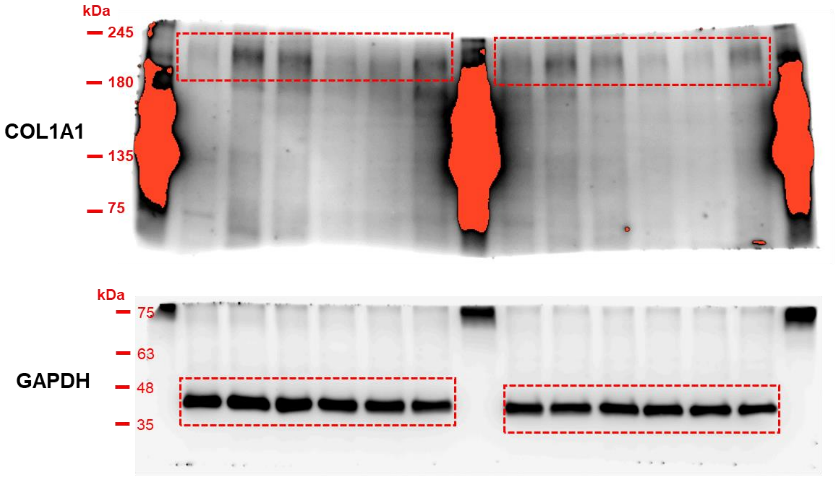

Supplementary Figure 5. Original immunoblotting data for Fig. 6C
